# Effect of vaccination against bovine respiratory disease (BRD) in a calf rearing unit in Finland

**DOI:** 10.1186/s13028-025-00808-7

**Published:** 2025-06-04

**Authors:** Katja Mustonen, Heidi Härtel, Heli Simojoki

**Affiliations:** 1 Department of Production Animal Medicine, University of Helsinki, Paroninkuja 20, Saarentaus, FI-04920 Finland; 2 HKFoods Finland Oy, PL 50, Turku, FI-20521 Finland; 3 Department of Agricultural Sciences, Faculty of Agriculture and Forestry, University of Helsinki, Koetilantie 5, Helsinki, FI-00790 Finland

**Keywords:** Antimicrobial treatment, Bovine respiratory disease, BRD, Morbidity

## Abstract

Bovine Respiratory Disease (BRD) is the main health concern in calf-rearing units. It is a major cause of increased antibiotic use and the leading cause of morbidity and mortality in calves. Vaccination protocols against BRD for calf-rearing units are difficult to implement in practice. The aim of this study was to evaluate the effect of the vaccination protocol including intranasal and subcutaneous vaccinations on mortality, antibiotic treatment rate, and average daily gain (ADG). The vaccination protocol consisted of intranasal BRD vaccination when the calves arrived at the rearing unit at the age of two to four weeks and two subcutaneous BRD vaccinations at two and three months of age. Mortality, antibiotic treatments, and ADG were recorded and evaluated from arrival until six months of age. The batches that arrived at the rearing unit prior to the beginning of the trial were used as the historic control group. Altogether, 740 vaccinated and 914 unvaccinated calves were enrolled to the study. A total of 88 calves (5.3%) died or were euthanized during the study period, of which 29 (32.9%) were vaccinated and 59 (67.1%) unvaccinated. In the logistic mixed model, the vaccination protocol decreased mortality (odds ratio 0.57, *P* = 0.036). The deaths occurred mostly during the pre-weaning period and only six calves died after weaning. During the study period, 1592 (96.3%) of the calves were treated with antibiotics at least once. In 90% of the courses, respiratory infections were the cause of antibiotic therapy. The mean antibiotic treatment rate for vaccinated calves (2.3 courses/calf, standard deviation [SD] 1.2) was lower than unvaccinated calves (2.4 courses/calf, SD 1.3) (*P* = 0.003). The average daily weight gain during the entire study period did not differ between the groups (vaccinated calves 1.08 kg/d, SD 0.12; unvaccinated calves 1.09 kg/d, SD 0.13). The vaccination protocol used in this study decreased the odds ratio for mortality but did not affect ADG. The difference in number of antibiotic treatments used for BRD was clinically negligible. A limitation of the study design is the interpretation of the effect of the historical control group which may affect the results through seasonal variation.

## Findings

Bovine Respiratory Disease (BRD) is an infectious respiratory disease in cattle and the main health concern in calf-rearing units. It is a major cause of increased morbidity and mortality in calves and leads to economic losses and increased use of antibiotic treatments [1–3]. Although vaccination against respiratory pathogens is a frequent practice, there is conflicting evidence of its efficacy on the reduction of morbidity and mortality in calves with BRD [4, 5]. For vaccinations to be effective on calf rearing farms appropriate immunization programs need to be initiated at the dairy farms, which is challenging to implement in practice.

This field study was conducted on one commercial calf-rearing unit in Finland. Before starting the study, the farm veterinarian wanted to ensure that the herd had the infections indicated for the vaccines. Blood samples were collected from 20 calves and serum examined in the Centre of Diagnostics Solutions (Boxmeer, The Netherlands) with enzyme-linked immunosorbent assay (ELISA) tests. Antibodies against coronavirus, bovine respiratory syncytial virus (BRSV), parainfluenza 3 virus (PI3), *Pasteurella multocida*, *Mannheimia haemolytica* and *Mycoplasma bovis* were detected, all pathogens associated with BRD.

**Fig. 1:**
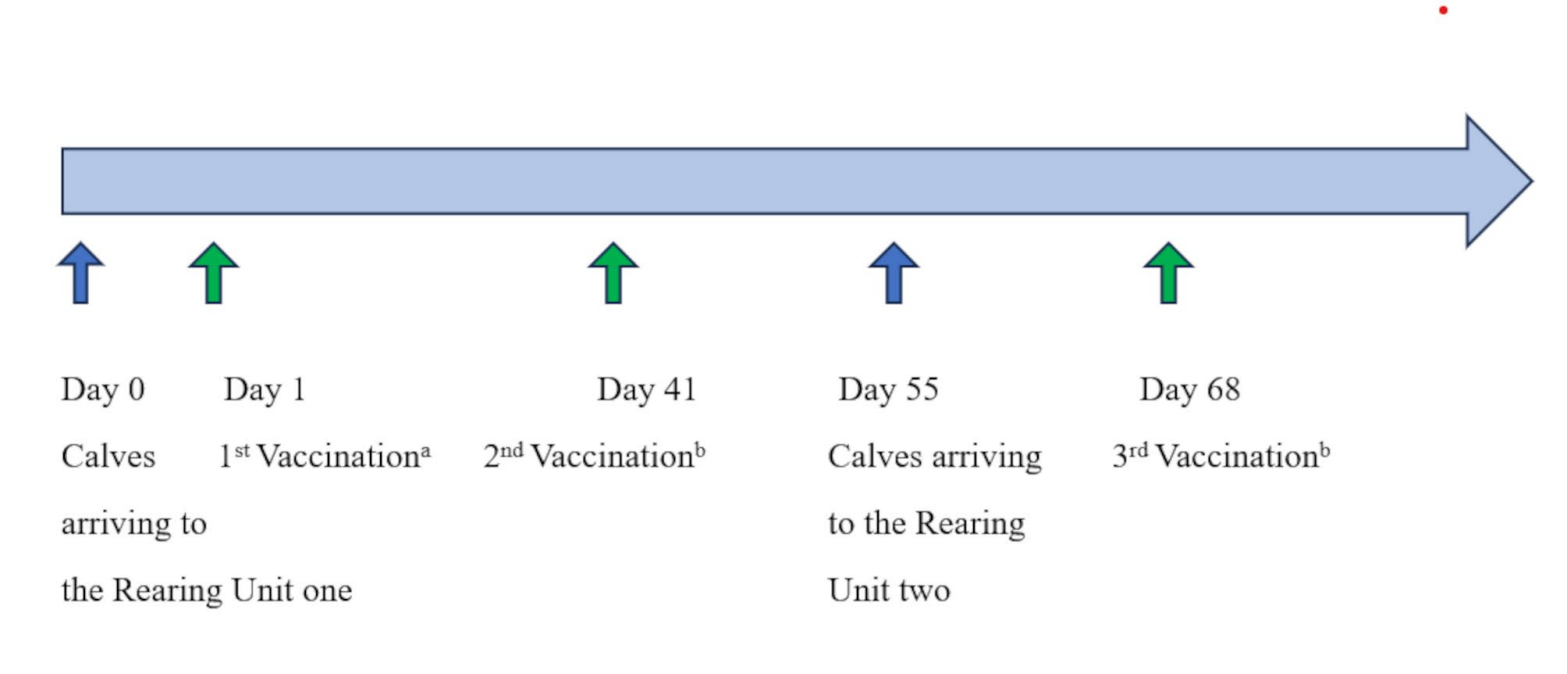
Vaccination protocol, vaccines used and the route of administration in the field study conducted in one calf rearing unit. **a** Bovilis RSP Live Vet, Intervet International B.V. (Bovine Respiratory Syncytical virus, parainfluenza-3 virus). **b** Bovilis Bovipast RSP, Intervet International B.V. (Bovine Respiratory Syncytical virus, parainfluenza-3 virus, *Mannheimia haemolytica*) in intranasal sc subcutaneus

The calves entering the rearing unit were collected from multiple dairy farms and transported to Rearing Unit1 at two to four weeks of age. Twelve batches of calves entered Rearing Unit1, in groups of approximately 60 calves per batch (mean 61.7, range 52–72 calves), between 22 March and 22 June 2021. They were transported to Rearing Unit2 after the milk-feeding period at the age of 2.5 months. At the age of six months, the calves were transported to the Finishing Unit. Rearing Unit1 worked with an All-In-All-Out system by compartments, and the batches of calves were kept isolated from each other. The vaccine protocol included one intranasal BRD vaccination which was twice boosted with subcutaneous vaccination (Fig. 1).

The control group, consisting of 14 batches of calves (approximately 60 calves per batch, mean 65.3, range 60–72 calves), entered the Rearing Unit 1 between 11 December 2020, and 15 March 2021. They were collected from Finnish dairy farms and transported to Rearing Unit1, Rearing Unit2 and to the Finishing Unit at the same age as the study group. The control group did not receive any vaccinations during the study period.

The calves were weighed at arrival to Rearing Unit1, at transport from Unit1 to Unit2 as well as at transport from Unit2 to the Finishing Unit. Throughout the study period, treatments and mortality of the calves were observed and recorded daily by the animal caretaker. Information on all medications, reasons for treatments, and deaths were gathered from the database of Centralized Healthcare Register for Finnish Cattle Herds (Naseva) during the study period. Sick calves were treated by the animal caretaker according to the instructions of the farm veterinarian. Antibiotic treatment for BRD (Table 1) was started if a calf had respiratory symptoms (cough, rapid or difficult breathing) and two of the following signs: body temperature > 39.7 °C, loss of appetite or depression.

**Table 1:** Type of antibiotics used in a calf-rearing unit during the 6-month rearing period

	Antibiotic	Vaccinated	Unvaccinated
1st treatment			
	Oxytetracycline	645 (89.33%)	789 (90.48%)
	Benzylpenicillin	68 (9.42%)	74 (8.49%)
	Tulathromycin	8 (1.11%)	7 (0.80%)
	Sulfa-trimethoprim	1 (0.14%)	2 (0.23%)
	Total	722 (100%)	872 (100%)
	Targeted against BRD	653 (90.44%)	796 (91.28%)
2nd treatment			
	Oxytetracycline	350 (66.41%)	419 (59.86%)
	Benzylpenicillin	5 (0.95%)	7 (1.00%)
	Tulathromycin	167 (31.69%)	262 (37.43%)
	Sulfa-trimethoprim	5 (0.95%)	12 (1.72%)
	Total	527 (100%)	700 (100%)
	Targeted against BRD	512 (97.00%)	661 (94.43%)
≥ 3rd treatment			
	Oxytetracycline	366 (87.35%)	559 (87.34%)
	Benzylpenicillin	19 (4.53%)	29 (4.53%)
	Tulathromycin	32 (7.64%)	41 (6.41%)
	Sulfa-trimethoprim	2 (0.48%)	11 (1.72%)
	Total	419 (100%)	640 (100%)
	Targeted against BRD	384 (91.65%)	560 (87.50%)

There were 1654 calves in the study, of which 740 were vaccinated and 914 were unvaccinated. BRD was the most common reason for antibiotic use

All statistical analyses were performed in Stata/MP 17 (StataCorp LP, Texas, USA). A logistic mixed regression model was used to study the association between vaccination and death of the calf during the study period. The association between vaccination and the number of antibiotic treatments per calf and ADG were studied using linear mixed regression models. The clustering of the data was taken account by using the calf batch as a group variable in all models. The number of antibiotic treatments used in the models were categorized into one, two, or three or more courses. The nominal variables sex and breed and the continuous variable age at arrival were included in all models (Table 2). The number of antibiotic treatments per calf was included in the models with the outcome variables mortality and ADG. A P-value < 0.05 was considered statistically significant.

**Table 2:** Characteristics of the calves included into the study, total *n* = 1654 SD standard deviation

	Vaccinated (*n* = 740)	Unvaccinated (*n* = 914)
Arrival age, days mean (SD)	16.2 (5.5)	17.3 (6.1)
The age of the calf at the time of the first course of antibiotics, days mean (SD)	29.1 (0.57)	26.5 (0.56)
Sex		
Heifer	204	248
Bull	536	666
Breed		
Finnish Ayrshire	156	197
Holstein	254	322
Aberdeen Angus mixed	42	63
Dairy beef crosses	264	305
Other breeds	24	27

SD Standard deviation

Altogether, 745 vaccinated and 918 unvaccinated calves were enrolled in the study. According to the Summary of the Product Characteristics for the vaccine, the onset of immunity is one week after vaccination with the intranasal vaccine [6]. Therefore, nine calves (five vaccinated and four unvaccinated) that died or were euthanized within seven days after arrival were excluded from the study. After that, the study consisted of 1654 calves; 740 vaccinated and 914 unvaccinated calves (Table 2). During the study period, 1592 (96.3%) of the calves were treated with antibiotics at least once and they were included in the statistical models.

A total of 5.3% (*n* = 88) calves died during the study period (Table 3). Most of the deaths occurred during the milk-feeding period; only six calves died or were euthanized after weaning (five vaccinated and one unvaccinated). Additionally, two vaccinated and six unvaccinated calves were slaughtered premature at the age of five to six months instead of normal slaughter at age 18 to 20 months due to poor growth. These calves were categorized as dead in the analysis of this study. Vaccinated calves had a lower odds ratio (OR) for mortality than unvaccinated calves in the logistic mixed regression model (Table 4).

**Table 3:** Calves that died, were euthanized or slaughtered prematurely during the study period

	Vaccinated	Unvaccinated
Number of calves	29 (32.9%)	59 (67.1%)
Mean age at time of death (days)	52.6 (SD 47)	46.7 (SD 50.4)

SD Standard deviation

**Table 4:** The association between the mortality and vaccination status of the calves (*n* = 1592) in one rearing-unit

	Odds ratio^*^	SD	*P*-value	95% Conf. Interval	
Vaccination	0.573	0.152	0.036	0.340	0.965
Arrival age	0.991	0.020	0.663	0.954	1.031
Sex					
Heifer	ref.				
Bull	1.753	0.020	0.082	0.932	3.299
Breed					
Finnish Ayrshire	ref.				
Holstein	0.925	0.274	0.792	0.518	1.651
Aberdeen Angus mixed	0.598	0.343	0.370	0.195	1.838
Dairy beef crosses	0.658	0.222	0.214	0.340	1.273
Other breeds	0.847	0.557	0.801	0.233	3.076
			Wald test for breed *P* = 0.705		
Number of antibiotic treatments					
None	ref.				
One course	0.282	0.111	0.001	0.131	0.609
Two courses	0.194	0.075	0.000	0.091	0.414
Three or more courses	0.089	0.037	0.000	0.039	0.202
	Wald test for number of antibiotic treatments *P* = 0.001				

^*^Logistic mixed regression model

All antibiotic treatments were administered intramuscularly. The number of antibiotic treatments given to an individual calf varied from one to seven courses. The mean antibiotic treatment rate was 2.3 courses/calf (SD 1.2) for vaccinated calves and 2.4 courses/calf (SD 1.3) for unvaccinated calves. The reason for antibiotic treatments was respiratory infection in 90% of the courses, and antibiotics were used according to the national recommendations (Table 1) [7]. In the linear mixed regression model to study the association between the number of antibiotic treatments (1–7) and vaccination status of the calves, vaccination reduced the number of antibiotic treatments used for calves (Table 5).

**Table 5:** The association between the number of antibiotic treatments^**^ and vaccination status of the calves (*n* = 1592)

	Coefficient^*^	Std error	*P*-value	95% Conf. Interval	
Vaccination	-0.211	0.098	0.031	-0.403	-0.019
Arrival age	-0.007	0.004	0.115	-0.017	0.002
Sex					
Heifer	ref.				
Bull	-0.102	0.071	0.154	-0.242	0.038
Breed					
Finnish Ayrshire	ref.				
Holstein	-0.177	0.077	0.021	-0.327	-0.027
Aberdeen Angus mixed	-0.502	0.127	0.001	-0.751	-0.254
Dairy beef crosses	-0.790	0.082	0.001	-0.951	-0.628
Other breeds	-0.400	0.170	0.018	-0.733	-0.069
			Wald test for breed *P* = 0.001		

^*^Linear mixed regression model. ^**^The number of antibiotic treatments was 1–7

ADG during the milk-feeding period was 0.88 kg/d (SD 0.17) in vaccinated calves (*n* = 717) and 0.97 kg/d (SD 0.17) in unvaccinated calves (*n* = 862). After weaning, ADG was 1.71 kg/d (SD 0.21, *n* = 710) and 1.77 kg/d (SD 0.24, *n* = 855), for vaccinated and unvaccinated calves, respectively. For the entire study period, ADG was 1.08 kg/d (SD 0.12) in vaccinated calves (*n* = 710) and 1.09 kg/d (SD 0.13) in unvaccinated calves (*n* = 855). Although ADG during the milk-feeding period was lower in vaccinated calves than in unvaccinated calves (coef − 0.080, *P* = 0.001), vaccination status of the calves did not influence ADG either at the time after weaning (coef − 0.05, *P* = 0.16) or during whole study period (coef − 0.006, *P* = 0.60). In the statistical model of associations of ADG during the entire study period and variables, every antibiotic treatment (1, 2 or ≥ 3) decreased ADG by approximately 50 g/day (Wald test *P* = 0.004).

In this study, mortality (5.3%) was slightly higher, as compared to a previous study in Finland (4.5%) [8]. In a field study conducted previously in Finland, intranasal vaccination of calves at arrival to the rearing unit did not affect mortality [5], contradictory with the current study. In contrast to the current trial, which involves two subcutaneous immunization boosters, the calves in the previous study in similar conditions received only one dose of the intranasal vaccination. In our study the death rate may have been impacted by the subcutaneous vaccinations’ targeting of *M. haemolytica* in addition to BRSV and PI3.

Vaccinations did not remove a need of the antibiotic treatments for BRD and the difference in the number of antibiotic courses is clinically negligible although it was statistically significant. In Danish herds there were no correlation between the use of antibiotics and respiratory vaccine sales numbers [2]. In the previous study in Finland, vaccination also had no significant effect on antibiotic use in calves [5]. The antibiotic treatment rate for BRD during the milk-feeding period in our study was remarkably high, which suggests that most of the calves were diseased. No metaphylactic treatments are allowed in Finland, thus all medications were based on respiratory symptoms of individual calves, and they were treated individually with intramuscular antibiotics. The historical control group, used in the current study (14 batches of calves), may be problematic, and affect the results as disease pressure can vary, for example, according to the different seasons. In Finland outside temperatures fluctuate which puts a burden on ventilation. In commercial calf rearing units using all-in/all-out system, there are practical difficulties in implementing other field study designs especially when a live inactivated intranasal vaccine is used. Vaccinated calves can shed vaccinal agents to non- vaccinated calves up to eleven days.

Although vaccinated calves had lower ADG during the milk-feeding period than unvaccinated calves, vaccination had no effect on ADG after weaning or during the whole study period. In contrast, intranasal vaccine alone had a growth-enhancing effect in a previous Finnish study [5]. It is possible that the lower mortality in the vaccination group, a different vaccination protocol, and a different type of control group may explain the difference in results [5].

The vaccination protocol used in this study reduced the OR for mortality but did not influence ADG. The difference in the number of antibiotic treatments used for BRD was of limited relevance, however, any reduction of antimicrobial usage may reduce development of antibiotic resistance and therefore has a clear benefit for the One Health perspective. The economic benefit for the individual farmers depends on the cost of the vaccinations which differs between countries compared to the individual herd improvements resulting from vaccinations.

## Data Availability

The datasets used and analysed during the current study are available from the corresponding author on reasonable request.

## Declarations

### Prior publication

Data have not been published previously.
